# New retinal tack designs: an analysis of retention forces in human scleral tissue

**DOI:** 10.1007/s00417-020-04689-6

**Published:** 2020-04-29

**Authors:** Markus Schulze Schwering, Theo Oltrup, Kai Sinan Rückheim, Thomas Bende, Karl Ulrich Bartz-Schmidt, Martin Alexander Leitritz

**Affiliations:** 1 Centre for Ophthalmology, University Eye Hospital, Tübingen, Germany; 2 Section for Experimental Eye Surgery and Refractive Surgery, Centre for Ophthalmology, University Eye Hospital, Eberhard Karls University of Tübingen, Schleichstr. 12/1, 72076 Tübingen, Germany

**Keywords:** Retinal tack, Retention, Penetration, Disentanglement

## Abstract

**Purpose:**

The study aimed to construct a new retinal tack design with high retention forces to prevent spontaneous disentanglement in cases of complicated retinal surgery.

**Methods:**

Six new forms for the peak of a retinal tack were developed using computer-aided design (CAD); then a prototype was produced for each model. Finally, standardised design testing was conducted using human (ex vivo) sclera by logging 15 consecutive measurements for each model.

**Results:**

Seven different models underwent pull-out testing (six new models and the original tack model), but two tack models (Model 4, Model 5) failed to penetrate the human tissue. The highest pull-out forces (median) were measured for Model 3, followed by Model 6, Model 2 and Model 1. The original Heimann tack (Model H) was found to have the lowest retention forces.

**Conclusion:**

The different tack designs altered the penetration and holding forces. The retention forces of the proposed peak design led to a significant increase in the retention forces that were more than twice as high as those in the original Heimann Model.

## Introduction

Different repair methods are needed for retinal surgery. Retinal holes can be fixed using laser coagulation or cryopexy. Traditionally, retinal detachment was fixed using scleral buckling with or without drainage of subretinal fluid; later, pneumatic retinopexy and primary vitrectomy were used [1]. Barrie [1] debated about which surgical technique to choose to fixate the retina, depending on the location and size of the retinal breaks, the presence of media opacities, the presence of proliferative vitreoretinopathy and the surgeon’s ability. An actual multicentre study by Shu et al. showed high final success rates in cases with rhegmatogenous retinal detachment that underwent scleral buckling or pars plana vitrectomy [2]. In some exceptional cases, retinal tacks are useful as an adjunctive instrument to repair complicated retinal detachments [3–8], for example, in cases of re-detachment with tissue shrinking following severe proliferative diabetic retinopathy, giant retinal tears [9] or in traumatic lesions, such as ruptures [3]. Furthermore, tacks can be used to anchor epiretinal electrode arrays [10]. Several studies have evaluated the biocompatibility of retinal tacks [11–15], but no long-term follow-up examination of tissue changes has been conducted. Fixation of the retina or the electrodes, respectively, can be achieved by retinal tacks that fully penetrate the posterior coats of the retina, choroid and sclera. Clinically, we have used the retinal tacks in the Heimann Model (Model H).

**Fig. 1:**
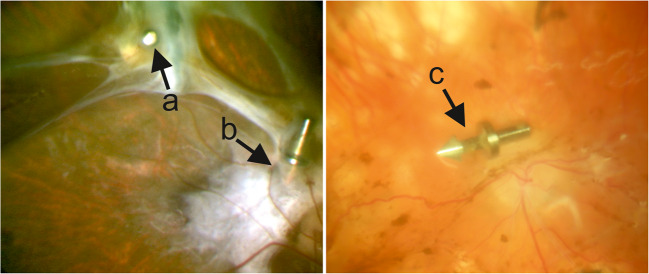
**(**Left) **a, b** retinal tacks fixating the retina in proliferative vitreoretinopathy (**a** fixated, **b** partially released); (right) **c** retinal tack after disentanglement (Heimann Model)

Unfortunately, in various cases, we observed spontaneous luxation of the tacks. In addition to oral reports of spontaneous disentanglement after fixation of retinal detachments (Communication with KU Bartz-Schmidt) (Fig. 1), published studies have described the set of problems associated with dislocated tacks and concomitant complications [16].

Thus, the present study aimed to develop a new design for the peak of a retinal tack with higher retention forces than is possible with the tacks in the original Heimann Model. The usability and anchorage of the retinal tack models were tested using human sclera.

## Material and methods

### Design and production of the retinal tack models using surgical steel

**Fig. 2:**
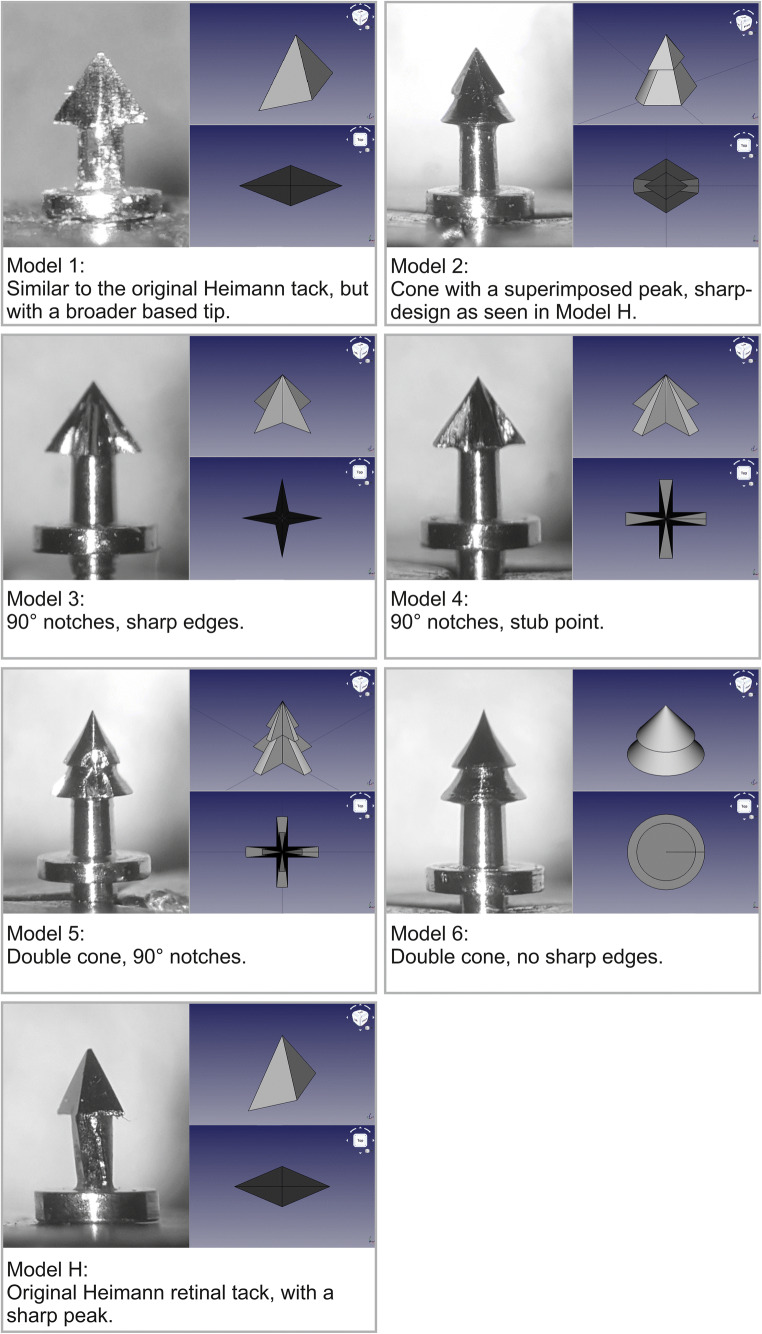
Retinal tack design versions. On the left side for each model, a photograph is shown. On the right side for each model, computer designs in two different perspectives (side, from top) are shown

Apart from the original Heimann retinal tack model (Fig. 2; Model H), six additional retinal tack models (Fig. 2, Models 1 to 6) were designed using computer-aided design (CAD) (AutoCAD®, Autodesk Inc., San Rafael, CA, USA). Each of the models had different peaks. Further details about the cone design for each of the models are written in Fig. 2. All of the tacks had a total length of 2.4 mm with a shaft length of 0.4 mm and a stabilisation plate with a diameter of 1 mm.

### Sclera preparation

The human sclera used in this study was from the Eye Hospital Biobank, and it was obtained from eyes that were enucleated on the basis of other diseases (e.g. melanoma). The sclera samples in which the configuration was obviously normal were processed within 24 h after enucleation.

The specimens were prepared in the following way. Scleral tissue (15 mm diameter) was excised. We then placed the tissue on a polystyrene globe (25 mm diameter). The inner side of the scleral tissue (vitreous side) was positioned so that it was lying towards the globe. Retention tests were performed on the outer side of the sclera.

### General principles of the pull-out test

In material testing, the pull-out test is a standardised procedure (EN ISO 6 892-1) for metallic materials. A fixed specimen is stretched consistently under a low velocity and shock-free condition until it breaks. During this process, force in Newton (N) and changes in length in metre (m) are continuously measured. In this way, it is possible to determine the specific data cluster for different material grades in order to compare them.

**Fig. 3:**
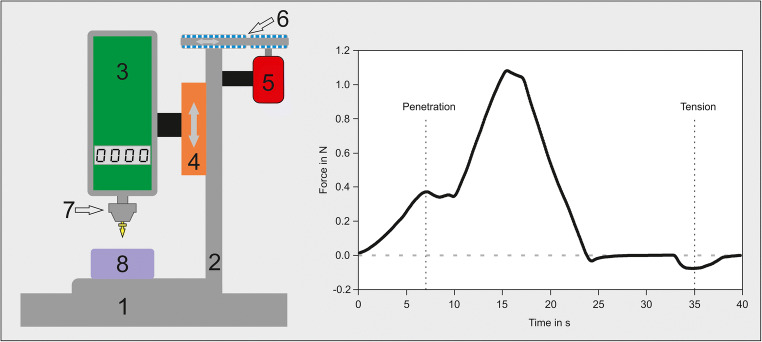
(Left) Measurement setting scheme: (1) desk; (2) stiff framework; (3) force gauge; (4) positioner; (5) stepping motor; (6) driving belt; (7) boring socket holding the retinal tack (yellow); and (8) test material. (Right) Standard example of the pull-out test: typical test frequency. The force before penetration increases (positive forces in Newton). Once first penetration is achieved, less force is needed before penetrating deeper with higher forces. The relaxing phase occurs after the tack is completely anchored in the tissue. Tensile forces are much lower than penetrating forces

The test setting is illustrated in Fig. 3 (left). The modified boring socket enabled us to clamp different tacks. Due to the stiff connection between the tack holder and the force gauge, it is possible to measure both the compressive and drag forces. The holding fixture for the sclera samples can be rotated after each measurement with a defined angle to avoid multiple insertions on the same localisation and to ensure that comparable initial conditions are used.

The following test sequence was used and resulted in measurement curves, as shown in Fig. 3 (right):

1. The retinal tack is pressed into the sclera model using a maximum force of 4 N (velocity of 0.14 mm/s) via a stiff connection with the force gauge in a vertical direction.
2. The tack remains in the sclera with a force of 1 N for 25 s in order to anchor it completely (dynamics/elasticity of the tested material).
3. The sclera and tack are unstressed for 3 s (pressure < 0.3 N).
4. The tack is removed backwards in a vertical direction with a velocity of 0.14 mm/s.
5. A maximum holding force (N) is noted.
6. The holding fixture for the silicone/sclera samples is turned to a new position, and the entire process is repeated.

### Statistics

The measurement data were analysed using JMP 13 statistical software (SAS Institute, Inc., Cary, NC, USA). The Wilcoxon signed rank test was performed to evaluate the changes and homogeneity, and the level of significance was set at *p* = 0.05. This study followed the tenets of the Declaration of Helsinki. Approval was obtained from the local ethics committee.

## Results

After completing the first step, which entailed theoretical planning using CAD, it was technically possible to produce all of the designed models using surgical steel.

For the test rows, probes of three different eyes were used. Tack Model 4 and Model 5 failed to reliably penetrate the human tissue because of very high penetration forces. Therefore, these tacks had to be excluded from further measurements.

Fifteen consecutive measurements (Table 1) were performed for each tack model. While performing the pull-out tests, the movement of the tacks was observed; if the objects were displaced (e.g. because of possible misalignment in the tissue), the measured value was excluded.

**Table 1:** Results of the pull-out tests using human sclera as the test tissue

Pull-out test in N*	Model 1	Model 2	Model 3	Model 6	Model H
Mean (*n* = 15)	0.541	0.711	1.00	0.604	0.363
Standard deviation	0.299	0.506	0.381	0.417	0.147
Minimum	0.142	0.07	0.332	0.132	0.174
Maximum	1.012	1.906	1.745	1.363	0.566
Median	0.518	0.607	0.964	0.649	0.337
No. of excluded measurements	1	1	1	4	2

For tack Model 4 and Model 5, no measurements were possible due to penetration failure

**Fig. 4:**
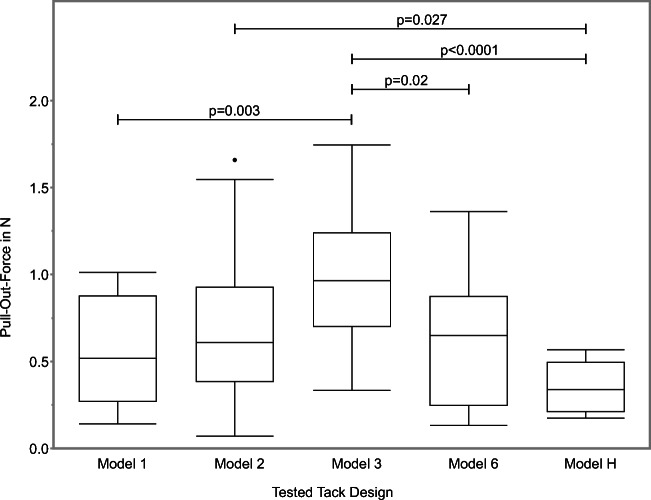
Pull-out forces (*y*-axis) for the tested tack designs. The box and whisker diagrams show the 5% and 95% quantiles (whiskers) and 25% and 75% quantiles (box) and medians (marked by a line). The horizontal lines show a comparison of the pairs and the resulting *p* values

The Wilcoxon signed rank test showed that the pull-out forces were significantly higher for Model 3 than Model 1 (*p* = 0.003), Model 6 (*p* = 0.02) and Model H (*p* < 0.0001) (Fig. 4). Moreover, the retention values were significantly higher for Model 2 than Model H (*p* = 0.027). In all the other comparisons between the different models, no statistically significant differences were found.

## Discussion

The present study’s findings show that the alternative tack designs had a significantly higher anchorage in comparison with the original Heimann tack (Model H). Of the six developed models, Model 4 and Model 5 failed to penetrate the donor tissue, but the other four models showed higher anchorage values than Model H (the original tack model).

The original research question sought to determine how to construct a new design for a retinal tack with high retention forces to prevent spontaneous disentanglement after fixation within special surgical situations, for example, for anchoring epiretinal electrodes [10]. The Heimann retinal tack (Model H) has a peak with sharp edges, so it cuts the underlying tissue by the width of its peak. Therefore, the holding forces of the barbed hook could be reduced. Because we observed retinal tack disentanglement in clinical settings, we wanted to create new peak forms for the retinal peak, as shown with the design alternatives (Model 1 to Model 6). We presumed that a cone-shaped retinal tack might penetrate the scleral model better and it would displace the underlying tissue without cutting it in order to maintain higher retention forces. The results from our experiment show that a certain degree of sharp edges or a sharp peak seemed to be necessary to penetrate human scleral tissue.

On the material side, the tack’s peak seems to be the most important factor in terms of the forces that interact with the tissue. Once placed, the fixation should be as durable as possible, and it should also be resistant to partially increasing forces, as seen in proliferative vitreoretinopathy or scar formations. Therefore, the maximal pull-out force needed to remove the tack from the tissue is the relevant experimental parameter.

A tack with sharp edges might be an alternative to the original Heimann tack with a single sharp peak. The standard Heimann tack (Model H) has sharp edges, similar to a jagged arrowhead. Model 3 has sharp edges and 90° notches. Its statistically significant difference (*p* < 0.0001) becomes obvious in the pull-out test: The retention forces were found to be more than twice as large in Model 3 than in Model H (original Heimann tack).

Furthermore, the tack material should be changed for use within an in vivo setting. For our test purposes, we worked with tacks made of surgical steel. To reduce potential risks (e.g. warming during MRI examinations), the material should be switched to titanium or plastic. In the latter case, the small peaks and notches must be produced with sufficient stability.

Clinical tests with titanium tacks, similar to Model 3, in human eyes, seem to be justified. Against the background of missing studies comparing retinal tacks with conventional vitrectomy or scleral buckling, a clinical examination focused on these areas would be of interest.

Photographs of penetrated scleral tissue obtained using electron microscopy might help to evaluate if the tacks are cutting the tissue or just displacing it. This would correspond to the potentially evoked trauma in subjects’ retina, choroid and sclera tissues.

## Study limitations

This study had some limitations. The tests were not blinded, and they were performed in ex vivo sclera samples. We, therefore, cannot foresee the long-term effects or the transferability of our results to a clinical situation. Another weakness is that we were unable to compare the tissue changes after removing a standard Heimann retinal tack and a Model 3 to evaluate the resulting tissue damage.

## Conclusions

Using our described experimental set-up, we showed that different tack designs resulted in changes in the penetration and holding forces within human sclera tissue. A new design (Model 3) led to retention forces that were more than twice as effective as that of the original Heimann tack (Model H). Our tests encourage future clinical use of a newly designed titanium retinal tack model in human eyes with the possibility of increased holding forces that could lead to less spontaneous disentanglement, as observed by ourselves or as reported in published work (e.g. by Lewis [16] or Mansour [17]).

## Data Availability

The datasets analysed during the current study are available from the corresponding author on reasonable request.
